# Dairy Processing and Breast Cancer: A Hypothesis-Generating Framework Linking Bioactive Lipid Derivatives to Epidemiological Heterogeneity

**DOI:** 10.1016/j.advnut.2026.100682

**Published:** 2026-06-15

**Authors:** Silia Ayadi, Julio Buñay, Chloé Gressein, Laly Pucheu, Ben Allal, Philippe de Médina, Sandrine Silvente-Poirot, Marc Poirot

**Affiliations:** 1 Team INOV: "Cholesterol metabolism and therapeutic innovations", Centre de Recherche en Cancérologie de Toulouse, INSERM, CNRS, University of Toulouse, Toulouse, France; 2 Team DIAD: “Dose individualization of anticancer drugs," Centre de Recherche en Cancérologie de Toulouse, INSERM, CNRS, University of Toulouse, Toulouse, France; 3 Institut Claudius Regaud, Institut Universitaire du Cancer-Oncopole, Toulouse, France

**Keywords:** dairy products, breast cancer, food processing, bioactive lipids, oxysterols, sterol metabolism, lipid oxidation, ultraprocessed foods, nutritional epidemiology, IGF-1

## Abstract

The relationship between dairy consumption and breast cancer risk remains complex and heterogeneous, with epidemiological studies reporting divergent associations depending on the type of dairy product and its degree of processing. Although nutritional composition has traditionally been considered the main explanatory factor, emerging evidence suggests that technological processing may also influence the biological properties of dairy foods. In this narrative review, we propose a hypothesis-generating framework that integrates epidemiological findings with experimental data on lipid oxidation and sterol-derived metabolites produced during dairy processing. In particular, cholesterol oxidation products and other bioactive lipid derivatives have been shown in experimental models to modulate pathways involved in inflammation, oxidative stress, and hormone signaling. However, direct evidence linking dietary exposure to these compounds and breast cancer risk in humans remains limited. Recent research highlights that food processing may generate a wide range of bioactive molecules beyond conventional nutrients, some of which may act at low concentrations and influence key regulatory pathways. These include both potentially deleterious and protective sterol-derived metabolites, whose balance may be affected by technological processing. Identifying such compounds, quantifying their dietary exposure, and understanding their biological effects represent important challenges for future research. Although current evidence remains insufficient to establish causal relationships, this framework supports the hypothesis that processing-related molecular changes may contribute to the heterogeneity observed in epidemiological studies.

## Graphical abstract

**Figure:**
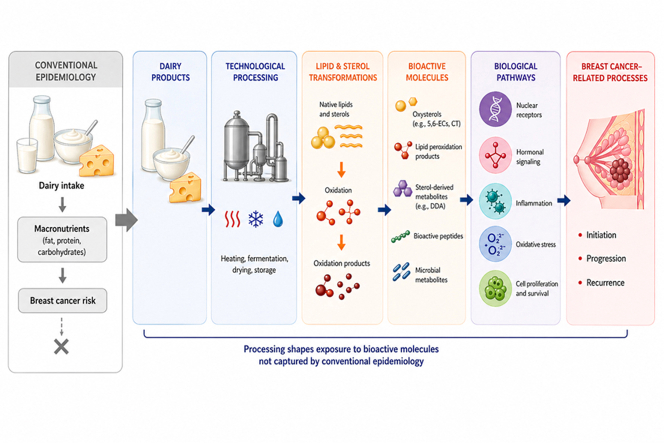

**Statement of significance**

This review proposes a novel integrative framework linking technological processing of dairy products to the formation of bioactive lipid and sterol-derived molecules that may influence breast cancer-related pathways. By integrating nutritional epidemiology, food chemistry, and molecular mechanisms, this work provides a new perspective for explaining the heterogeneity of epidemiological findings beyond traditional nutrient-based approaches.

## Introduction

Breast cancer remains the most commonly diagnosed cancer among women worldwide and a leading cause of cancer-related mortality, with incidence continuing to rise in many regions [1]. Lifestyle factors, including diet, are increasingly recognized as important determinants of breast cancer risk and progression [2,3]. Among dietary exposures, dairy products have received considerable attention due to their complex composition, which includes macronutrients, hormones, and a wide range of bioactive lipids. However, epidemiological studies examining dairy consumption and breast cancer have yielded inconsistent findings, with reports of neutral, inverse, or positive associations depending on the population, tumor subtype, and type of dairy product consumed [[4], [5], [6]].

**FIGURE 1:**
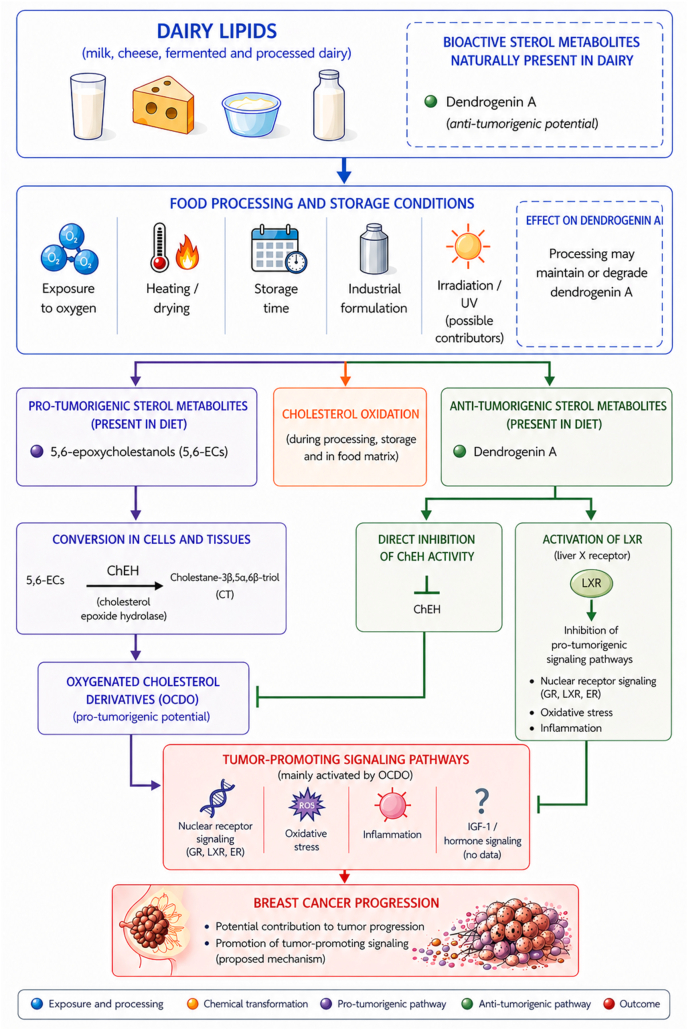
Formation of cholesterol oxidation products (oxysterols) and their proposed biological effects. Cholesterol oxidation may occur during food processing and storage, leading to the formation of oxysterols. Among these, 6-oxo-cholestan-3*β*,5α-diol (OCDO) has been shown to exert tumor-promoting effects in preclinical breast cancer models and has been reported at elevated levels in patients with breast cancer. Several oxysterols have been suggested to modulate inflammation, oxidative stress, and hormone receptor signaling in experimental systems. However, their role in human disease and their relationship to dietary exposure remain uncertain and warrant further investigation. This figure summarizes proposed mechanisms and does not imply causality. ChEH, cholesterol epoxide hydrolase; ER, estrogen receptor; GR, glucocorticoid receptor; IGF-1, insulin-like growth factor 1; LXR, liver X receptor.

A major limitation of current research is the frequent treatment with dairy foods as a homogeneous exposure, despite substantial variability in their composition and processing. Milk, fermented products, cheeses, and highly processed dairy foods differ not only in fat content and fermentation status, but also in their exposure to technological processes such as heating, drying, and storage. These processes may alter lipid structure and promote the formation of oxidized lipids and sterol-derived metabolites [[7], [8], [9], [10]]. Experimental studies suggest that such compounds, including oxysterols and related cholesterol derivatives, can modulate nuclear receptor signaling, inflammatory pathways, and hormonal regulation, all of which are involved in breast carcinogenesis [[11], [12], [13]]. Conversely, fermentation processes may generate bioactive peptides and microbial metabolites that could exert beneficial effects through interactions with the gut microbiota and immune system [14]. More broadly, these observations suggest that dairy products should not be considered solely as sources of nutrients, but as complex matrices whose molecular composition may be dynamically modified by technological processing (Figure 1).

**FIGURE 2:**
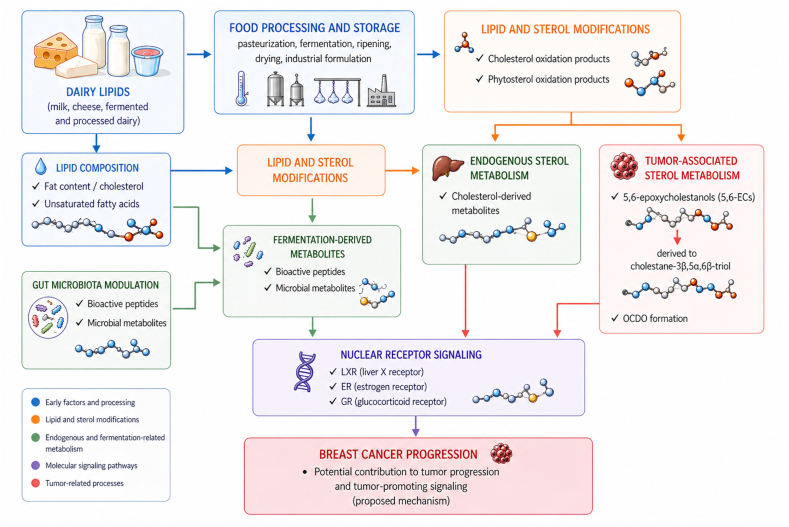
Conceptual framework linking dairy processing, lipid transformations, and cancer-related pathways. This schematic illustrates how different levels of dairy processing may influence lipid composition and the generation of bioactive compounds, including oxidation products and fermentation-derived metabolites, which have been proposed to interact with biological pathways relevant to carcinogenesis. These relationships are based on experimental and observational evidence and are presented as a hypothesis-generating framework. The diagram does not imply causal relationships, and the relevance of these mechanisms in humans remains uncertain and warrants further investigation. ER, estrogen receptor; GR, glucocorticoid receptor; IGF-1, insulin-like growth factor 1; LXR, liver X receptor; OCDO, oncosterone.

Despite these mechanistic insights, the potential contribution of processing-induced changes in lipid and sterol composition has rarely been integrated into the interpretation of epidemiological findings. This gap may partly explain the persistent heterogeneity observed across studies, particularly when different categories of dairy products are pooled or insufficiently characterized. In addition, most epidemiological analyses do not capture qualitative differences in lipid oxidation status or sterol composition, and the relevance of these molecular changes to human exposure remains uncertain (Figure 2).

In this context, the present narrative review proposes a hypothesis-generating framework linking dairy processing, lipid and sterol transformations, and biological pathways relevant to breast cancer. The objectives are to *1*) critically synthesize epidemiological evidence linking dairy consumption to breast cancer risk, tumor subtype, and prognosis, with attention to differences according to dairy type; *2*) summarize current knowledge on lipid oxidation and sterol metabolism in dairy products, including the impact of technological processing; and *3*) explore how processing-related changes may contribute to the heterogeneity of observed associations. By integrating nutritional epidemiology, food science, and mechanistic data, this review aims to provide a more nuanced perspective on dairy-related exposures and to identify key directions for future interdisciplinary research. These considerations support the need to incorporate food processing and molecular composition into nutritional epidemiology, moving beyond nutrient-based approaches toward a more food-structure-oriented perspective [15].

## Methods

This narrative review was conducted using a structured literature search to summarize current evidence on the association between dairy product consumption and breast cancer risk, tumor subtype, and prognosis, with particular attention to the type of dairy product and the degree of food processing.

A literature search was performed in the PubMed/Medline database from inception to January 2026 to identify relevant articles published in English. This database was selected because of its broad coverage of biomedical, nutritional, and food science research. Search terms included combinations of keywords related to dairy products and breast cancer (e.g., “dairy,” “milk,” “yogurt,” “cheese,” “fermented dairy,” “high-fat dairy,” “ultraprocessed food,” “food processing,” “breast cancer”), as well as terms related to potential biological mechanisms [e.g., “IGF-1 (Insulin-like growth factor-1),” “hormones,” “lipid oxidation,” “oxysterols,” “cholesterol oxidation,” “phytosterol oxidation,” “nuclear receptors,” “microbiota,” “inflammation”].

The PubMed/Medline search focused on dairy foods and breast cancer yielded 135 records. Titles and abstracts were screened for relevance to dairy consumption and breast cancer incidence, subtype, prognosis, recurrence, or mortality. Relevant publications were subsequently evaluated in full text and complemented by additional references identified through citation tracking of selected articles. In total, 82 publications were retained and cited in the final manuscript, including studies identified through database searches and additional references identified through citation tracking and targeted searches of relevant mechanistic and food composition literature. These included epidemiological studies and meta-analyses addressing dairy intake or related dietary exposures in relation to breast cancer outcomes, as well as selected mechanistic, food composition, food processing, and methodological publications required to support the hypothesis-generating framework. Because this work was designed as a narrative review rather than a systematic review, no formal PRISMA-based screening process or independent dual-review selection was applied.

Eligible publications included prospective cohort studies, case–control studies, randomized controlled trials when available, and systematic reviews and meta-analyses examining dairy intake in relation to breast cancer incidence, subtype, survival, or recurrence. No restriction was applied on study size. Experimental studies were also considered when relevant to the discussion of biological mechanisms, particularly those investigating lipid oxidation products, sterol-derived metabolites, nuclear receptor signaling, or the effects of fermentation and food processing on dairy composition.

The primary focus of this review was dairy foods consumed as part of the human diet, including milk, yogurt, cheese, fermented dairy products, processed dairy foods, and ultraprocessed dairy products. Dairy-derived ingredients and supplements, such as whey protein powders, casein supplements, milk protein concentrates, and dairy protein isolates, were not considered as primary exposure categories because they are generally consumed outside the native dairy matrix and are not consistently assessed in nutritional epidemiology studies. However, mechanistic studies involving dairy proteins or protein fractions were considered when relevant to pathways discussed in this review, including IGF-1 signaling, insulin signaling, and processing-related biological effects.

Studies were selected based on relevance to the objectives of the review. Exclusion criteria included studies not addressing dairy consumption, studies focusing on nonbreast cancer outcomes, and publications lacking sufficient methodological detail or outcome reporting. Reference lists of selected articles were screened to identify additional relevant studies. Both studies reporting positive, inverse, or null associations were included to ensure a balanced synthesis of the evidence.

Given the substantial variability in dairy composition and processing, studies were not limited to total dairy intake but also included investigations evaluating individual dairy products, including milk, yogurt, cheese, and processed dairy foods. Particular attention was given to studies distinguishing between fermented and nonfermented products, low-fat and high-fat dairy, and minimally processed compared with processed or ultraprocessed foods. When available, studies using classification systems based on the degree of processing, including the NOVA framework [16], were considered.

In addition to epidemiological studies, reports describing food composition were reviewed to assess the potential formation of biologically active lipid derivatives during processing and storage. Databases compiling oxidized sterol concentrations in foods, such as the FooDOxS database [17], were examined to identify the presence of cholesterol oxidation products (COPs), phytosterol oxidation products (POPs), and related compounds in dairy products. Experimental studies investigating endogenous sterol-derived metabolites, including molecules produced during cholesterol metabolism in mammary cells, were also considered to inform the discussion of potential biological mechanisms.

This review was not designed as a formal systematic review and was not conducted according to PRISMA guidelines [18]. A narrative approach was selected because of the heterogeneity of available studies in terms of design, exposure definition, and outcome assessment. The objective was not to provide an exhaustive evidence synthesis but rather a structured narrative review integrating epidemiological findings, food composition data, food processing characteristics, and mechanistic evidence relevant to dairy products and breast cancer. The evidence was synthesized narratively, with emphasis on consistency across studies, biological plausibility, and potential sources of heterogeneity. Particular attention was given to key limitations of current epidemiological research, including measurement error in dietary assessment, insufficient classification of dairy products, limited information on food processing, and the lack of data on low-abundance bioactive compounds that may exert biological effects through signaling pathways. A further limitation of the available epidemiological literature is the lack of standardized definitions for dairy intake categories. Terms such as "high," "higher," or "low" intake frequently correspond to study-specific quantiles or exposure categories rather than harmonized consumption thresholds, which limits direct comparisons across studies.

## Biological Mechanisms Linking Dairy Intake, Processing, and Breast Cancer

The potential biological mechanisms linking dairy intake, food processing, and breast cancer are summarized in Figure 2.

The potential association between dairy consumption and breast cancer has been investigated for several decades, and several biological mechanisms have been proposed to explain both protective and adverse effects. Dairy products contain a complex mixture of nutrients and bioactive compounds, including proteins, lipids, hormones, vitamins, and minerals, whose effects on carcinogenesis may differ according to the type of dairy product, fat content, and processing conditions. The most frequently discussed mechanisms involve IGF-1 signaling, steroid hormones, lipid composition, calcium and vitamin D intake, and the effects of fermentation on gut microbiota and immune regulation.

### Hormonal and growth factor pathways

One of the most frequently proposed mechanisms linking dairy consumption to breast cancer risk involves the modulation of hormonal and growth factor pathways, particularly IGF-1, steroid hormones, and insulin signaling. These pathways play central roles in cell proliferation, differentiation, and apoptosis, and dysregulation of these processes is known to contribute to mammary carcinogenesis. Because dairy products contain biologically active compounds and may influence endogenous hormone levels, their potential impact on breast cancer development has been extensively investigated.

Milk consumption has been consistently associated with increased circulating concentrations of IGF-1 in both children and adults. IGF-1 is a peptide hormone involved in somatic growth and tissue development, but elevated circulating levels have been linked to increased risk of several cancers, including breast cancer. Prospective pooled analyses have shown that individuals in the highest categories of circulating IGF-1 concentrations have a greater risk of developing breast cancer compared with those in the lowest categories, and that this association appears to be stronger for estrogen receptor (ER)-positive tumors [19,20].

Dairy intake may influence IGF-1 levels through several mechanisms. Milk naturally contains IGF-1; however, more importantly, it may stimulate endogenous production of IGF-1 in the liver through increased intake of protein and essential amino acids. Controlled feeding studies and observational data have shown that consumption of milk or dairy protein can increase circulating IGF-1 concentrations compared with lower or nondairy intake [21,22]. Because IGF-1 promotes cell proliferation and inhibits apoptosis, chronic elevation of this hormone has been proposed as a plausible pathway linking high dairy intake to increased cancer risk, although epidemiological findings remain inconsistent.

In addition to IGF-1, dairy products may contain steroid hormones derived from pregnant cows, including estrogens, progesterone, and their metabolites. Estrogen exposure is a well-established risk factor for breast cancer, particularly for hormone-dependent tumors. Milk from pregnant cows contains higher concentrations of estrogens, and it has been hypothesized that dietary exposure to these hormones could influence circulating hormone levels in humans. Some authors have suggested that modern dairy production practices, which often involve milking cows during pregnancy, may increase the hormonal content of milk compared with historical conditions [23]. However, the actual contribution of dietary estrogens from dairy products to systemic hormone levels appears to be small, and most epidemiological studies have not demonstrated a strong association between dairy intake and circulating estrogen concentrations.

Insulin signaling may also play a role in the relationship between dairy consumption and breast cancer. Diets rich in energy and certain macronutrients can influence insulin secretion and insulin resistance, which in turn affect the IGF-1 axis and other metabolic pathways involved in carcinogenesis. Some studies have suggested that high intake of dairy protein may increase insulin secretion, although the clinical relevance of this effect remains unclear. Hyperinsulinemia and insulin resistance have been associated with increased breast cancer risk, particularly in postmenopausal women, suggesting that metabolic factors may partly mediate the association between diet and cancer development [[24], [25], [26]].

Despite the biological plausibility of these mechanisms, epidemiological evidence linking dairy intake to breast cancer through hormonal pathways remains inconsistent. Some cohort studies report positive associations, others show null results, and several suggest inverse relationships for specific dairy products. These discrepancies may reflect differences in dairy type, fat content, fermentation, or processing level, which are rarely considered in studies focusing on total dairy intake. Moreover, hormonal effects may vary according to menopausal status, body composition, and tumor subtype, further complicating interpretation.

Because hormonal and growth factor pathways represent only 1 aspect of the biological effects of dairy consumption, additional mechanisms related to lipid composition and fat content have also been proposed and are discussed in the following section.

### Fat content and lipid-related mechanisms

The lipid composition of dairy products has long been considered a potential factor influencing breast cancer risk. Dairy fat contains a complex mixture of SFAs, MUFAs and PUFAs, cholesterol, phospholipids, and minor bioactive lipids, all of which may affect metabolic and inflammatory pathways involved in carcinogenesis. Because high intake of dietary fat has been associated with obesity, insulin resistance, and chronic low-grade inflammation, several studies have examined whether consumption of high-fat dairy products is related to breast cancer incidence or prognosis. However, results remain inconsistent, suggesting that the relationship between dairy fat and breast cancer is more complex than initially assumed.

Early epidemiological studies reported positive associations between total fat intake and breast cancer risk, leading to the hypothesis that saturated fat consumption could promote mammary tumor development. A pooled analysis of prospective cohort studies found only weak associations between total fat intake and breast cancer, with no clear evidence that saturated fat alone strongly increased risk [27]. Large cohort studies have similarly suggested that the relationship between dietary fat and breast cancer is modest and may vary according to menopausal status and other metabolic factors [28]. These findings suggest that the role of fat intake may be mediated partly through adiposity and metabolic factors rather than direct carcinogenic effects.

Dairy products contribute substantially to saturated fat intake in many populations, and several studies have evaluated whether high-fat dairy consumption is associated with breast cancer risk. Some prospective studies have reported that intake of high-fat dairy products is associated with increased mortality after breast cancer diagnosis, whereas low-fat dairy intake shows no such association. In a cohort of breast cancer survivors, women in the highest compared with lowest categories of high-fat dairy intake after diagnosis had increased breast cancer-specific mortality, suggesting that fat content may influence disease progression [29]. These observations have led to the hypothesis that saturated fats have been shown to influence tumor-related pathways in experimental systems, through effects on inflammation, insulin resistance, or estrogen metabolism, although causality cannot be established from observational data.

In addition to SFAs, dairy fat contains cholesterol, which plays a central role in cell membrane structure and steroid hormone synthesis. Cholesterol metabolism has been increasingly implicated in cancer biology, as tumor cells require cholesterol for membrane synthesis and signaling. Experimental and mechanistic studies have shown that cholesterol and its metabolites can regulate proliferation, migration, and survival of breast cancer cells through multiple signaling pathways. However, epidemiological evidence linking dietary cholesterol intake to breast cancer risk remains limited and inconsistent, although recent reviews highlight the importance of cholesterol metabolism and sterol-derived signaling in tumor development [11,13,30,31].

Dairy fat also contains several bioactive lipids that may exert beneficial effects. Conjugated linoleic acid (CLA), naturally present in ruminant fat, has been shown in experimental models to inhibit tumor growth and modulate immune function. Some authors have proposed that CLA and other ruminant-derived fatty acids could partly explain the neutral or inverse associations observed between dairy intake and breast cancer in epidemiological studies [32,33]. In addition, milk fat globule membrane components, including sphingolipids and phospholipids, have been reported to influence cell signaling and apoptosis, although their relevance for human cancer risk remains uncertain [34].

An additional aspect that has received increasing attention is the possibility that lipid composition may be altered during food processing. Heat treatment, drying, and storage can promote oxidation of cholesterol and unsaturated fatty acids, generating biologically active derivatives that may influence inflammatory and hormonal pathways. Because these oxidation products are more likely to be present in processed or long-shelf-life dairy products, differences in technological processing may partly explain the heterogeneous associations observed between dairy intake and breast cancer. These processing-related mechanisms are discussed in more detail in the following sections.

### Calcium, vitamin D, and micronutrients

Dairy products are major dietary sources of calcium and frequently contribute substantially to vitamin D intake in populations where milk is fortified. Because both calcium and vitamin D have been implicated in the regulation of cell proliferation, differentiation, and apoptosis, their intake has been proposed as a possible mechanism linking dairy consumption to breast cancer risk. Experimental studies have shown that calcium may inhibit cell growth and promote differentiation in epithelial tissues, whereas vitamin D exerts antiproliferative and proapoptotic effects through activation of the vitamin D receptor. These observations have led to the hypothesis that higher intake of calcium and vitamin D could reduce risk of hormone-dependent cancers, including breast cancer.

Early epidemiological studies suggested that women in the highest categories of calcium intake had a lower breast cancer incidence than those in the lowest categories. In a large prospective analysis from the Nurses’ Health Study, higher dietary calcium intake was linked to reduced risk of breast cancer, particularly among premenopausal women [35]. Subsequent cohort studies reported similar findings, although the magnitude of the association varied across populations. In the Nurses’ Health Study, women in the highest compared with lowest categories of calcium and vitamin D intake from both dietary and supplemental sources had a modestly lower risk of premenopausal breast cancer, whereas no clear association was observed for postmenopausal breast cancer [36].

Vitamin D has attracted particular interest because of its role in regulating gene transcription through the vitamin D receptor, which is expressed in normal and malignant breast tissue. Experimental studies indicate that vitamin D signaling may inhibit tumor growth, reduce inflammation, and modulate immune responses [37]. Observational studies have reported inverse associations between circulating vitamin D levels and breast cancer risk, although results are not consistent across all cohorts. In the Cancer Prevention Study II Nutrition Cohort, higher intake of calcium and vitamin D was associated with lower risk of premenopausal breast cancer, but no significant association was observed in postmenopausal women [38].

Because dairy products are among the main dietary sources of calcium, it has been suggested that the inverse association observed in some studies between dairy intake and breast cancer could reflect the effect of calcium rather than dairy products themselves. However, studies comparing calcium from dairy and nondairy sources have yielded inconsistent results, and meta-analyses have concluded that the association between calcium intake and breast cancer risk is modest and not consistently observed across populations [39,40].

Another factor complicating interpretation is that dairy products differ widely in their content of micronutrients depending on processing, fortification, and fermentation. For example, vitamin D content varies considerably between countries according to fortification policies, and fermented dairy products may contain different levels of bioavailable minerals compared with nonfermented products. In addition, high intake of calcium may influence the absorption of other nutrients and hormones, which could indirectly affect cancer risk.

Overall, the available evidence suggests that calcium and vitamin D may contribute to a modest reduction in breast cancer risk, particularly in premenopausal women, but findings are not consistent and do not fully explain the heterogeneous associations observed with dairy intake. These limitations indicate that other components of dairy products, including those modified by fermentation or food processing, may also play a role. In particular, fermentation can alter nutrient composition and generate bioactive compounds capable of influencing immune function and hormone metabolism, which are discussed in the following section.

### Fermentation, microbiota, and bioactive peptides

Fermented dairy products differ from nonfermented dairy foods not only in taste and texture but also in their biochemical composition and potential physiological effects. Fermentation involves the action of microorganisms that modify proteins, lipids, and carbohydrates, producing metabolites that may influence host metabolism, immune function, and hormonal regulation. Because of these changes, fermented dairy products such as yogurt, kefir, and certain cheeses have been proposed to exert different effects on cancer risk compared with nonfermented dairy foods. Several epidemiological studies have reported inverse associations between fermented dairy intake and breast cancer, suggesting that fermentation-related mechanisms may contribute to protective effects [4,41].

One of the most frequently discussed mechanisms involves the modulation of gut microbiota. Fermented dairy products contain live bacteria or bacterial metabolites that can influence the composition and activity of intestinal microbiota. Increasing evidence indicates that the gut microbiome plays an important role in systemic inflammation, immune regulation, and metabolism of steroid hormones, all of which are relevant to breast cancer development. Certain intestinal bacteria participate in the enterohepatic circulation of estrogens by producing enzymes that deconjugate estrogen metabolites, thereby affecting circulating hormone levels. Alterations in microbiota composition could therefore influence exposure of breast tissue to biologically active estrogens [14,42].

Fermented dairy products may also exert anti-inflammatory effects. Chronic low-grade inflammation has been implicated in the development of several cancers, including breast cancer, particularly in postmenopausal women. Probiotic bacteria present in fermented dairy foods can modulate immune responses and reduce production of proinflammatory cytokines. In experimental models, lactic acid bacteria have been shown to enhance immune surveillance and inhibit tumor growth, although the relevance of these findings for human cancer risk remains uncertain [43].

Another potential mechanism involves the production of short-chain fatty acids and other microbial metabolites during fermentation. These compounds may influence gene expression, cell differentiation, and apoptosis. Fermentation can also generate bioactive peptides derived from milk proteins, some of which have been reported to possess antioxidant, anti-inflammatory, or antiproliferative properties. Although most evidence for these effects comes from experimental studies, they provide biological plausibility for the inverse associations observed in some epidemiological investigations [14].

Several cohort studies and meta-analyses have suggested that fermented dairy intake may be associated with lower breast cancer risk. In pooled analyses of prospective studies, participants in the highest compared with lowest categories of yogurt and cultured dairy product consumption showed a reduced breast cancer risk in some populations, whereas nonfermented dairy products showed no clear association [4,41]. Differences in dietary patterns, fermentation methods, and types of fermented foods consumed may contribute to these discrepancies.

Fermentation may also influence the nutritional composition of dairy products by modifying lipid structure and reducing lactose content, potentially affecting metabolic responses. In addition, fermented dairy foods often undergo different technological processes compared with ultraprocessed dairy products, which may lead to differences in the formation of oxidation products or additives. These factors suggest that the degree of processing and the type of technological treatment could influence the biological effects of dairy products beyond their basic nutrient content.

Because fermentation can modify both the microbiological and chemical properties of dairy foods, it represents an important variable when interpreting epidemiological studies on dairy intake and breast cancer. However, most cohort studies classify dairy products only into broad categories and rarely distinguish between fermented, minimally processed, and highly processed foods. This limitation has led to increasing interest in the potential role of chemical changes induced by food processing, including lipid oxidation and sterol modification, which are discussed in the following section.

In addition to fatty acid composition, technological processing may also induce oxidation of lipids and sterols present in dairy fat.

### Oxidized sterols and lipid oxidation products generated during dairy processing: potential implications for breast cancer

COPs (oxysterols) have attracted increasing attention as potential mediators linking food processing to biological effects [44]. These compounds can be formed during thermal processing, storage, and exposure to oxygen, leading to structurally diverse molecules with distinct biological activities. More broadly, lipids present in dairy products, including cholesterol, phytosterols, and unsaturated fatty acids, are susceptible to oxidation during technological processes, generating COPs, POPs, and lipid peroxidation products [7,8,11,31,45,46].

These oxidized lipids have been detected in a variety of dairy products, particularly after high-temperature treatments or prolonged storage [7,8,47,48]. Experimental studies suggest that some of these compounds may influence cellular pathways involved in inflammation, oxidative stress, and cell proliferation [[11], [12], [13],^44^,[49], [50], [51]], raising the possibility that food processing could modify the biological properties of dairy products. However, most available evidence derives from in vitro and animal models, and their relevance to human health remains to be clarified.

Among COPs, 5,6-epoxycholestanols and their metabolites have attracted particular interest [11,31]. These compounds can be converted into cholestane-3*β*,5α,6*β*-triol (CT), an intermediate in the biosynthetic pathway leading to the formation of oncosterone (OCDO) [50]. OCDO has been shown to promote tumor growth in preclinical models of breast cancer and has been reported at elevated levels in patients [11,12,52], providing mechanistic insights into cholesterol-derived signaling pathways. However, several important limitations should be acknowledged. Importantly, OCDO is not a direct product of cholesterol autoxidation but results from enzymatic conversion of CT [52]. Although some of its upstream intermediates may arise from cholesterol oxidation processes, including those occurring during food processing, the formation of OCDO itself relies on endogenous enzymatic pathways. This observation raises the possibility that such metabolites could be present in mammary secretions and potentially in milk, although this remains to be experimentally demonstrated. In addition, the complexity of cholesterol metabolism makes it difficult to disentangle the respective contributions of diet, metabolism, and disease-related processes [44].

In addition to conventionally studied oxysterols, increasing attention has been given to a broader spectrum of cholesterol-derived metabolites that exert biological effects at low concentrations, particularly through modulation of nuclear receptor signaling pathways [11,12,49,53]. Among these, dendrogenin A (DDA) has been identified as an endogenous metabolite with tumor-suppressive properties in experimental models [8,11,13,44,[53], [54], [55], [56], [57], [58], [59], [60], [61]]. Notably, de Medina et al. [61] demonstrated that DDA can prevent the development of breast tumors in immunocompetent mouse models, thus providing evidence of its chemopreventive role in vivo. The anticancer properties of DDA were further confirmed in human tumors, including patient-derived xenografts in vivo [57,59,62,63]. More recent studies have further explored its mechanisms of action, including its involvement in intercellular communication processes mediated by extracellular vesicles [54,55]. In particular, these studies show that DDA induces sustained antitumor responses and has been associated with immune-mediated protection against tumor recurrence in preclinical models.

In addition, DDA has been identified in several mammalian species, including humans, mice, and cattle, suggesting that it may be conserved across mammals [31,61]. Interestingly, it has been detected in tissues enriched in epithelial cells, including mammary gland samples, which are directly involved in milk production [61]. This observation raises the possibility that such metabolites could be present in mammary secretions and potentially in milk, although this remains to be experimentally demonstrated. However, whether DDA is effectively secreted into milk, at what concentrations, and whether it remains stable during dairy processing are currently unknown. Therefore, although this hypothesis is biologically plausible, it remains to be experimentally demonstrated.

**FIGURE 3:**
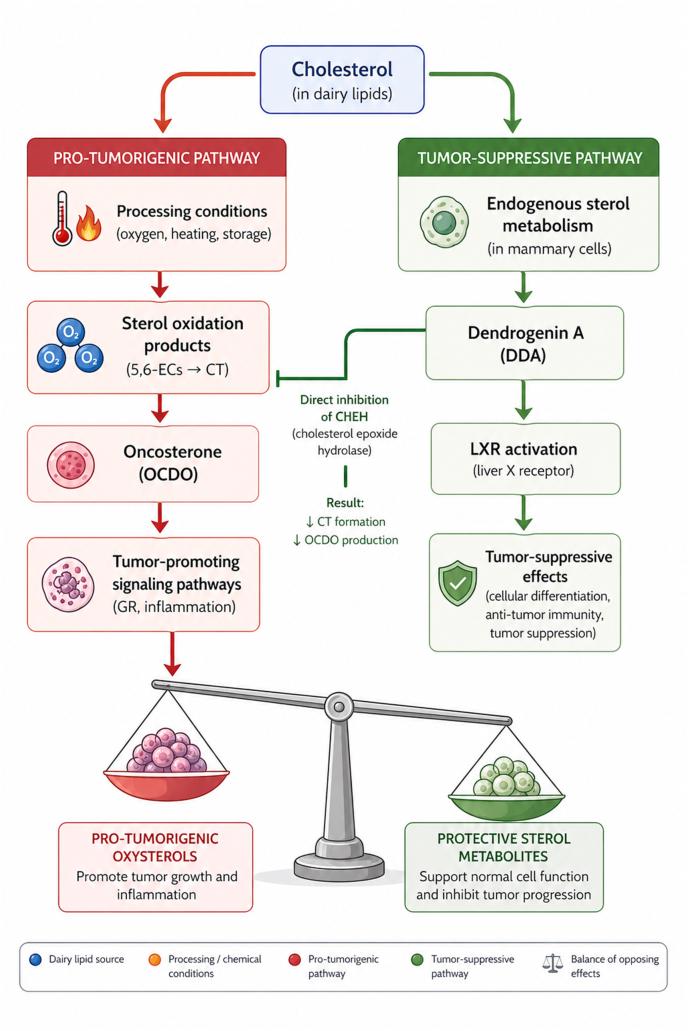
Hypothetical balance between cholesterol-derived metabolites with divergent biological effects. This schematic illustrates a proposed balance between metabolites such as OCDO and dendrogenin A (DDA), which have been associated with tumor-promoting and tumor-suppressive effects, respectively, in experimental models. The relative contribution of these pathways in humans, and their modulation by diet or food processing, remains uncertain. This representation is conceptual and intended to generate hypotheses rather than demonstrate causal relationships. CT, cholestane-3β,5α,6β-triol; EC, 5,6-epoxycholestanols; GR, glucocorticoid receptor; OCDO, oncosterone

These findings highlight the existence of cholesterol-derived metabolites with potentially opposing biological activities (Figure 3), acting at concentrations distinct from those of conventional nutrients. However, their relevance to human physiology, their presence in food matrices, and their modulation by dietary exposure or food processing remain largely unexplored. As a result, although these compounds provide important mechanistic insights, their role in human disease should be interpreted with caution.

Recent compositional databases, such as FooDOxS, provide quantitative data on oxidized sterols across a wide range of foods and highlight the influence of processing and formulation on their levels [17]. However, epidemiological studies rarely incorporate such information, and no large cohort study has specifically examined the association between dietary exposure to oxidized sterols and breast cancer risk or prognosis. Importantly, these mechanisms should not be interpreted as direct explanations for epidemiological associations between dairy intake and breast cancer risk. Taken together, these observations suggest that oxidized lipids represent biologically plausible candidates linking dairy processing to cancer-related mechanisms. At the same time, the potential contribution of endogenous bioactive sterol-derived metabolites, possibly present in animal-derived foods, warrants further investigation. However, their role in human disease, and particularly their relevance in the context of dietary exposures, remains uncertain and warrants further investigation. Understanding whether technological processing leads to meaningful differences in exposure to these compounds may help explain the heterogeneous associations observed in epidemiological studies.

## Milk Consumption and Breast Cancer Risk

Milk is the most commonly consumed dairy product worldwide and has therefore been the focus of numerous epidemiological studies investigating the relationship between dairy intake and breast cancer. However, findings remain inconsistent, with some studies reporting neutral associations, others suggesting increased risk, and several indicating possible protective effects depending on the population studied, the level of consumption, and the characteristics of the tumor. These discrepancies may reflect differences in study design, dietary patterns, and biological mechanisms related to hormonal exposure, growth factor signaling, and fat content.

Early cohort and case–control studies generally reported no strong association between milk intake and breast cancer risk. A meta-analysis of prospective studies including >1 million participants found that total dairy intake, including milk, was not significantly associated with breast cancer incidence, although moderate inverse associations were observed in some subgroups [4]. Similar results were obtained in later meta-analyses, which concluded that milk consumption does not appear to be a major determinant of breast cancer risk, but that heterogeneity between studies remains substantial [4,6].

One explanation for the inconsistent results is that the association between milk intake and breast cancer may depend on menopausal status. Several cohort studies have reported that milk consumption is inversely associated with premenopausal breast cancer, whereas results for postmenopausal breast cancer are weaker or null. In a prospective study from the Nurses’ Health Study, women in the highest categories of milk and dairy product intake had a reduced risk of premenopausal breast cancer, but no significant association was observed after menopause [35]. Hormonal differences between premenopausal and postmenopausal women, as well as variations in body composition and metabolic factors, may partly explain these findings.

Another important source of heterogeneity is tumor subtype. Breast cancer is a biologically diverse disease, and associations with dietary factors may differ according to hormone receptor status. In pooled analyses of cohort studies, milk intake was not associated with overall breast cancer risk, but differences were observed when tumors were classified according to ER status [5]. These findings are consistent with the hypothesis that different biological mechanisms may be involved in different tumor subtypes.

The potential role of hormonal and growth factor pathways has often been proposed to explain the association between milk consumption and breast cancer. Milk intake has been shown to increase circulating concentrations of IGF-1, which is involved in cell proliferation and inhibition of apoptosis. Elevated IGF-1 levels have been associated with increased risk of breast cancer in prospective studies, suggesting a plausible mechanism linking milk consumption to tumor development [20]. In addition, milk from pregnant cows contains small amounts of estrogens and progesterone, and it has been hypothesized that dietary exposure to these hormones could influence hormone-dependent cancers, although the clinical significance of this exposure remains uncertain [23].

Dose–response analyses have also suggested that the association between milk intake and breast cancer may not be uniform across intake levels. In meta-analyses of prospective studies, moderate milk consumption was generally not associated with increased risk, although substantial heterogeneity between studies was observed, and results varied according to population characteristics and study design [4,6].

Overall, current epidemiological evidence indicates that milk consumption is not consistently associated with breast cancer risk, but possible differences according to menopausal status, tumor subtype, and level of intake cannot be excluded. Because milk differs substantially from fermented dairy products in terms of microbial content and biochemical composition, these results have led to increasing interest in studies examining fermented dairy foods separately, which are discussed in the following section.

## Fermented Dairy Products and Breast Cancer Risk

Fermented dairy products, including yogurt, cultured milk, kefir, and certain fresh cheeses, differ from nonfermented dairy foods in both their microbial content and biochemical composition. Fermentation modifies proteins, lipids, and carbohydrates through the action of microorganisms, producing metabolites that may influence host metabolism, immune responses, and hormone regulation. Because of these differences, several epidemiological studies have examined fermented dairy products separately from total dairy intake, and some have suggested that these foods may be associated with a lower risk of breast cancer. Meta-analyses of prospective studies generally report neutral or inverse associations between fermented dairy consumption and breast cancer. In a meta-analysis including cohort and case–control studies, the highest compared with lowest categories of yogurt and fermented dairy intake were associated with a modest reduction in breast cancer risk, although heterogeneity between studies was substantial [4]. More recent meta-analyses of prospective studies similarly indicate that associations may vary according to the type of dairy product, with fermented dairy tending toward neutral or inverse associations in some populations [4,6,41]. These results suggest that fermentation-related changes in dairy composition may influence the relationship with breast cancer.

One of the most frequently discussed mechanisms involves the modulation of gut microbiota. Fermented dairy products contain live bacteria or bacterial metabolites that can influence the composition and activity of intestinal microbiota. Increasing evidence indicates that the gut microbiome plays an important role in systemic inflammation, immune regulation, and metabolism of steroid hormones, all of which are relevant to breast cancer development. Certain intestinal bacteria participate in the enterohepatic circulation of estrogens by producing enzymes that deconjugate estrogen metabolites, thereby affecting circulating hormone levels. Alterations in microbiota composition could therefore influence exposure of breast tissue to biologically active estrogens [14,42].

Fermented dairy products may also exert anti-inflammatory effects. Chronic low-grade inflammation has been implicated in the development of several cancers, including breast cancer, particularly in postmenopausal women. Probiotic bacteria present in fermented dairy foods can modulate immune responses and reduce production of proinflammatory cytokines. In experimental models, lactic acid bacteria have been shown to influence host immune function, although the relevance of these findings for human cancer risk remains uncertain [43].

Another potential mechanism involves the production of short-chain fatty acids and other microbial metabolites during fermentation. These compounds may influence gene expression, cell differentiation, and apoptosis. Fermentation can also generate bioactive peptides derived from milk proteins, some of which have been reported to possess antioxidant, anti-inflammatory, or antiproliferative properties. Although most evidence for these effects comes from experimental studies, they provide biological plausibility for the inverse associations observed in some epidemiological investigations [14].

Several cohort studies have also suggested that cultured or fermented dairy intake may be associated with lower breast cancer risk in some populations. Results from Swedish cohorts suggest that associations may differ according to dairy product type. Fermented dairy products have been associated with a lower risk of certain breast cancer subtypes, whereas nonfermented milk intake has shown positive associations with hormone receptor-positive tumors. These findings further support the importance of distinguishing dairy products according to their composition and processing characteristics rather than considering total dairy intake alone [41,64]. Differences in dietary patterns, fermentation methods, and types of fermented foods consumed may contribute to the discrepancies observed across studies [65].

Fermentation may also influence the nutritional composition of dairy products by modifying lipid structure and reducing lactose content, potentially affecting metabolic responses. In addition, fermented dairy foods often undergo different technological processes compared with ultraprocessed dairy products, which may lead to differences in the formation of oxidation products or additives. These factors suggest that the degree of processing and the type of technological treatment could influence the biological effects of dairy products beyond their basic nutrient content.

Overall, current evidence suggests that fermented dairy products are not associated with increased breast cancer risk and may be inversely associated in some populations. However, the number of studies examining individual fermented foods remains limited, and most epidemiological investigations do not distinguish clearly between different types of fermented dairy products. Further research is needed to determine whether the observed associations are related to fermentation itself, to differences in nutrient composition, or to variations in food processing. The role of cheese and other dairy products with higher fat content is discussed in the following section.

## Cheese, Dairy Fat, and High-Fat Dairy Products

Cheese and other high-fat dairy products represent an important source of saturated fat and cholesterol in many diets, and their potential role in breast cancer has been investigated in several epidemiological studies. Unlike milk, cheese is a heterogeneous category that includes fresh cheeses, fermented cheeses, and aged or hard cheeses, which differ substantially in fat content, microbial composition, and technological processing. Most epidemiological studies, however, group all cheese types together, making it difficult to determine whether specific products are associated with breast cancer risk.

Meta-analyses examining cheese consumption generally report no clear association with breast cancer incidence. In a meta-analysis of prospective studies, cheese intake was not significantly related to breast cancer risk, and results were similar when total dairy products were considered [4]. More recent meta-analyses have confirmed that dairy intake, including cheese, is not consistently associated with breast cancer risk, although substantial heterogeneity exists between studies [4,6]. These findings suggest that cheese, despite its high-fat content, may not increase breast cancer risk, but the heterogeneity of cheese types limits interpretation.

The fat content of dairy products has been proposed as a potential explanation for differences observed across studies. High-fat dairy products contain larger amounts of SFAs and cholesterol, which may influence metabolic and inflammatory pathways involved in carcinogenesis. Some epidemiological studies have reported that women in the highest categories of dietary fat intake have an increased breast cancer risk, particularly in postmenopausal women, although the magnitude of the association is generally modest [66]. Because cheese contributes substantially to saturated fat intake in many populations, it has been hypothesized that consumption of full-fat dairy products in the highest intake categories could promote tumor development, but evidence remains inconsistent.

In contrast, several studies have suggested that dairy fat may not be uniformly harmful and may contain bioactive components with potential protective effects. Milk fat includes CLA, sphingolipids, and other minor lipids that have been shown in experimental studies to modulate immune function, inflammation, and cell proliferation. These compounds have been proposed to counterbalance some of the adverse metabolic effects of saturated fat, which could explain why epidemiological studies do not consistently show increased cancer risk with high-fat dairy intake [65,67].

The role of dairy fat may be particularly relevant after breast cancer diagnosis. In a prospective cohort of breast cancer survivors, higher intake of high-fat dairy products after diagnosis was associated with increased breast cancer-specific mortality, whereas low-fat dairy intake was not associated with poorer outcomes [29]. These findings suggest that fat content may influence tumor progression or recurrence, possibly through effects on inflammation, insulin resistance, or hormone metabolism. However, observational studies cannot establish causality, and confounding by overall dietary pattern or body weight cannot be excluded.

Another limitation of the current literature is the lack of detailed classification of cheese types and processing methods. Hard cheeses, soft cheeses, processed cheese, and fresh cheeses differ in fat concentration, fermentation, salt content, and storage conditions, which may influence the formation of oxidation products or other bioactive compounds. Because most cohort studies rely on food frequency questionnaires with limited detail, it is difficult to evaluate whether technological processing modifies the association between cheese consumption and breast cancer.

Overall, available evidence indicates that cheese consumption is not consistently associated with breast cancer risk, but differences according to fat content, processing, and type of cheese cannot be excluded. These limitations highlight the importance of considering the degree of food processing when evaluating dairy products. In recent years, increasing attention has been given to the potential role of processed and ultraprocessed foods in cancer risk, and this question is particularly relevant for dairy products that undergo extensive technological transformation, as discussed in the following section.

## Processed and Ultraprocessed Dairy Products and Breast Cancer

In recent years, increasing attention has been given to the potential role of food processing in chronic disease risk, including cancer [68]. The NOVA classification system distinguishes foods according to the extent and purpose of industrial processing, categorizing them as minimally processed, processed, or ultraprocessed [69]. Ultraprocessed foods are typically formulations made from refined ingredients, additives, and industrially modified substances, and they often undergo multiple technological steps that can alter their nutritional and chemical composition. Individuals in the highest categories of ultraprocessed food consumption have been reported to exhibit an increased risk of several chronic diseases, including obesity, cardiovascular disease, and cancer. In the NutriNet-Santé cohort, a higher proportion of ultraprocessed foods in the diet was associated with increased risk of overall cancer and breast cancer [70]. Similar associations have been reported in systematic reviews and meta-analyses, although heterogeneity between studies is substantial [71,72].

**FIGURE 4:**
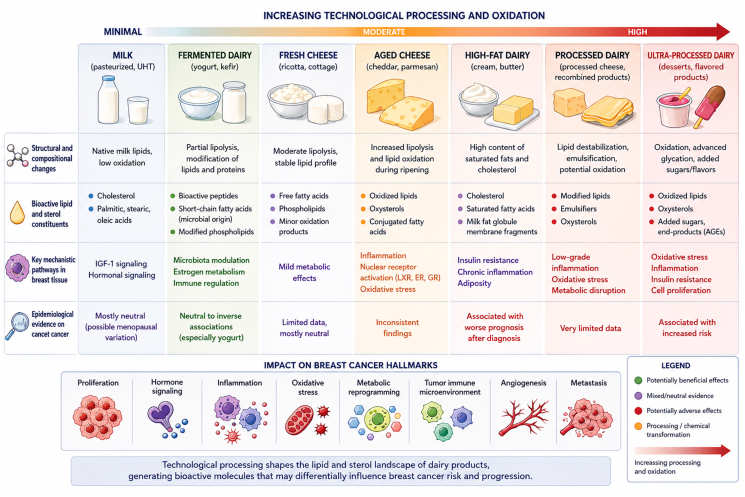
Classification of dairy products according to processing levels. Dairy products are categorized based on their degree of processing, from minimally processed to ultraprocessed, incorporating elements of existing classification systems. This framework is used to explore potential differences in nutritional composition and exposure to processing-related compounds. The classification does not imply differential health effects per se but serves as a basis for investigating associations observed in epidemiological studies. UHT, ultrahigh temperature.

Despite these observations, most studies evaluating ultraprocessed foods analyze total dietary intake and do not examine specific food groups in detail. Dairy products are often included within broader categories, making it difficult to determine whether ultraprocessed dairy products contribute to the observed associations. Dairy foods vary widely in their degree of processing, ranging from minimally processed milk to highly formulated products such as flavored yogurts, dairy desserts, processed cheese, and long-shelf-life milk products. These foods may differ not only in nutrient content but also in the presence of additives, sugar, emulsifiers, and compounds formed during technological processing. A classification of dairy products according to processing level is presented in Table 1. A schematic representation of dairy classification according to processing level is shown in Figure 4.

**TABLE 1:** Dairy products, processing level, lipid characteristics, and breast cancer evidence.

Dairy category	Processing level	Key lipid/sterol features	Mechanistic hypotheses	Epidemiological evidence
Milk	Minimal (pasteurization, UHT)	Cholesterol, saturated fat, IGF-1 stimulation; low oxidation when fresh	IGF-1 signaling, hormonal exposure	Mostly neutral; possible variation by menopausal status [[4], [5], [6]]
Fermented dairy	Moderate (fermentation)	Bioactive peptides, microbial metabolites, modified lipids	Microbiota modulation, estrogen metabolism, immune regulation	Neutral to inverse associations, especially yogurt [4,6]
Fresh cheese	Moderate, short storage	Moderate fat, limited oxidation	Similar to fermented dairy	Limited data; mostly neutral
Aged cheese	Fermentation + storage	Higher fat, potential lipid oxidation	Inflammation, oxysterol exposure	Inconsistent; mostly neutral [4]
High-fat dairy	Variable	High saturated fat, cholesterol	Insulin resistance, inflammation	Worse survival after diagnosis [28]
Processed dairy	Industrial processing	Modified lipids, emulsifiers, possible oxidation	Additives, lipid oxidation	Very limited data
Ultraprocessed dairy	Multiple processing steps	Oxidized lipids, sterol derivatives, added sugars	Oxidative stress, inflammation	Associated with increased cancer risk [66,67]
Oxidized sterols (cross-category)	Heat, storage, drying	7-ketocholesterol, 5,6-epoxysterols∗, sterane-3*β*,5α,6*β*-triol∗	Nuclear receptor signaling, tumor pathways	Not evaluated epidemiologically
Endogenous sterols (milk)	Physiological	Dendrogenin A	Tumor-suppressive pathways	Unknown (experimental evidence)

Abbreviations: IGF-1, insulin-like growth factor 1; UHT, ultrahigh temperature; ∗5,6-epoxysterols include 5,6-epoxycholestanols and 5,6-epoxyphytostanols; ∗sterane-3*β*,5α,6*β*-triol include cholestane-3*β*,5α,6*β*-triol and phytostane-3*β*,5α,6*β*-triol.

Sweetened and flavored dairy products may contain high amounts of added sugar, which has been associated with obesity and metabolic disturbances that can increase breast cancer risk. In addition, industrial processing can involve high-temperature treatments, spray-drying, and prolonged storage, all of which may promote oxidation of lipids and sterols. As discussed in previous sections, oxidation of cholesterol and unsaturated fatty acids can generate biologically active compounds capable of modulating inflammatory and hormonal pathways involved in tumor development. Because ultraprocessed dairy products are more likely to undergo such treatments, differences in processing level could influence the profile of bioactive molecules present in the final product.

Another potential factor is the use of food additives. Ultraprocessed dairy products may contain emulsifiers, stabilizers, artificial flavors, and other additives whose long-term effects on cancer risk are not fully understood. Experimental studies suggest that some additives can affect gut microbiota composition or intestinal permeability, which could indirectly influence inflammation and metabolic regulation. However, epidemiological data specifically linking additives in dairy products to breast cancer are currently lacking.

Few studies have examined individual dairy products within the context of ultraprocessed food consumption. In analyses of dietary patterns, sweetened dairy desserts and processed cheese have sometimes been included among ultraprocessed foods, but results are rarely reported separately for these items. Consequently, it remains unclear whether the association observed between ultraprocessed food intake and breast cancer is driven by specific food groups or reflects overall dietary patterns characterized by high energy density, low fiber intake, and high consumption of refined ingredients.

Overall, current evidence suggests that individuals in the highest categories of ultraprocessed food consumption may have an increased breast cancer risk, but the role of ultraprocessed dairy products specifically remains largely unexplored. Because industrial processing can modify lipid composition and generate oxidation products with biological activity, future studies should examine dairy foods according to their degree of processing. A better understanding of these differences may help explain the heterogeneous associations observed between dairy intake and breast cancer and may provide insight into potential mechanisms linking food processing to carcinogenesis.

In addition to changes in macronutrient composition, ultraprocessed dairy products may differ from minimally processed foods in their content of oxidized lipids and sterol derivatives generated during industrial transformation, but this aspect has not been evaluated in epidemiological studies.

## Dairy Intake and Breast Cancer Subtypes

Breast cancer is a heterogeneous disease composed of several biological subtypes that differ in prognosis, response to treatment, and risk factors. Tumors are commonly classified according to the expression of ERs, progesterone receptors, and human epidermal growth factor receptor 2 (HER2), as well as by molecular subtypes such as luminal, HER2-positive, and triple-negative breast cancer. Because these subtypes differ in their hormonal dependence and metabolic characteristics, it is plausible that dietary factors, including dairy intake, may influence their development differently. In recent years, several epidemiological studies have examined the association between dairy consumption and breast cancer risk according to tumor subtype, which may help explain the inconsistent results observed in studies analyzing breast cancer as a single outcome.

Pooled analyses of prospective cohort studies have provided some of the most informative data on this question. In large cohort studies and meta-analyses, total dairy intake was generally not associated with overall breast cancer risk, but differences emerged when tumors were classified according to hormone receptor status. In some analyses, consumption of certain dairy products in the highest compared with lowest intake categories was associated with reduced risk of specific tumor subtypes, whereas no clear association was observed for others, suggesting that dietary factors may influence breast cancer through mechanisms that differ according to hormone receptor expression [[4], [5], [6]]. These findings raise the possibility that nonhormonal mechanisms, such as inflammation, immune modulation, or metabolic signaling, may contribute to the development of certain breast cancer subtypes.

Other cohort studies have reported similar patterns. In a Swedish population-based study, women in the highest categories of cultured dairy intake had a reduced risk of breast cancer, with stronger associations for hormone receptor–negative tumors than for hormone receptor-positive tumors [41]. In contrast, some studies have suggested that milk intake may be positively associated with certain subtypes, particularly those less strongly related to estrogen exposure, although results are not consistent across populations [35]. These discrepancies may reflect differences in dietary habits, levels of consumption, or genetic and hormonal characteristics of the study populations.

The role of hormonal pathways may help explain subtype-specific associations. ER-positive tumors are strongly influenced by endogenous hormone levels, and factors that increase circulating estrogens or growth factors could promote their development. Because milk consumption has been associated with increased circulating IGF-1 concentrations, some authors have hypothesized that high intake of milk could influence risk of hormone-dependent tumors [20,22]. However, epidemiological evidence does not consistently support this hypothesis, and several cohort studies and meta-analyses have found no clear association between total dairy intake and ER-positive breast cancer [4,6]. These findings indicate that hormonal mechanisms alone cannot fully explain the relationship between dairy intake and breast cancer.

Inflammatory and metabolic pathways may be more relevant for certain subtypes, particularly triple-negative breast cancer, which is less dependent on estrogen signaling and more strongly associated with obesity and metabolic dysfunction. Fermented dairy products, which may influence gut microbiota and immune regulation, have been suggested to reduce inflammation and improve metabolic profiles, potentially contributing to lower risk of these subtypes. However, the number of studies specifically examining triple-negative breast cancer in relation to dairy intake remains limited, and results should be interpreted cautiously.

Another limitation of the current literature is that most studies classify dairy products only into broad categories and do not consider the degree of processing. Because processing can modify lipid composition, generate oxidation products, or introduce additives, it is possible that different types of dairy products may have distinct effects on different tumor subtypes. For example, minimally processed fermented dairy foods may differ biologically from highly processed dairy desserts or processed cheese, but these distinctions are rarely evaluated in epidemiological studies.

Overall, available evidence suggests that the association between dairy intake and breast cancer may vary according to tumor subtype, with some indications of inverse associations for hormone receptor–negative cancers and neutral associations for hormone receptor-positive tumors. However, data remain limited, and further studies with detailed classification of dairy products and tumor characteristics are needed to clarify these relationships. Understanding subtype-specific associations may help explain the heterogeneous results observed in the literature and may provide insight into the biological mechanisms linking dairy consumption, food processing, and breast cancer.

## Dairy Intake after Diagnosis and Breast Cancer Prognosis

In addition to its potential role in cancer development, dairy consumption may influence breast cancer prognosis after diagnosis. Survivorship has become an important area of research because improvements in screening and treatment have increased the number of women living with breast cancer. Dietary factors may affect recurrence, progression, and mortality through mechanisms involving hormonal regulation, inflammation, insulin resistance, and body weight. However, compared with studies on cancer incidence, relatively few investigations have examined the relationship between dairy intake and outcomes after breast cancer diagnosis, and available results are limited and sometimes inconsistent.

One of the most frequently cited studies on this topic is a prospective cohort analysis of breast cancer survivors that evaluated the association between dairy intake after diagnosis and mortality. In this study, higher consumption of high-fat dairy products was associated with increased breast cancer-related mortality, whereas intake of low-fat dairy products was not related to survival [29]. Women in the highest compared with lowest category of high-fat dairy intake had a significantly higher risk. These findings suggested that fat content rather than total dairy consumption might influence disease progression.

Similar observations have been reported in studies examining broader postdiagnosis dietary patterns, although results are not entirely consistent. In the Life After Cancer Epidemiology study, dietary patterns characterized by healthier overall intake were associated with better outcomes, whereas less favorable dietary patterns were associated with poorer prognosis [73]. Because high-fat dairy products contribute to saturated fat intake, these findings are compatible with the hypothesis that metabolic and inflammatory effects of dietary fat may influence breast cancer prognosis. However, the contribution of dairy products themselves is difficult to isolate from overall dietary patterns.

Several mechanisms have been proposed to explain why high-fat dairy intake could affect breast cancer progression. Diets high in saturated fat may promote weight gain and insulin resistance, which are associated with increased circulating insulin and IGF-1 levels. These hormones can stimulate tumor cell proliferation and inhibit apoptosis, potentially favoring tumor growth. In addition, adipose tissue is an important source of estrogen after menopause, and higher body fat mass is associated with increased estrogen production, which may promote hormone-dependent tumors. These pathways suggest that dietary factors influencing body weight and metabolism could affect breast cancer outcomes. Evidence from postdiagnosis cohort studies also suggests that lower intake of saturated and *trans* fat may be associated with improved survival after breast cancer diagnosis [74].

Despite these hypotheses, evidence regarding dairy intake after breast cancer diagnosis remains limited. Most studies have evaluated broad dietary patterns rather than specific dairy foods, and few have distinguished between fermented, minimally processed, or ultraprocessed dairy products. Moreover, food frequency questionnaires used in cohort studies often lack detailed information on technological processing, making it difficult to assess whether industrially processed dairy foods differ from traditional dairy products in their effects on prognosis.

Another limitation is that observational studies cannot fully account for confounding factors such as body weight, physical activity, treatment, and overall diet quality. Women who consume high amounts of high-fat dairy products may differ in many lifestyle characteristics from those who consume less, which may influence survival independently of dairy intake.

Overall, current evidence suggests that total dairy consumption after breast cancer diagnosis is not clearly associated with prognosis, but high intake of high-fat dairy products may be linked to poorer survival in some studies. Because the number of investigations is limited and detailed information on processing level is rarely available, further research is needed to determine whether specific types of dairy products influence recurrence or mortality. Recent reviews of diet and breast cancer prognosis also conclude that evidence remains mixed, particularly for fat intake and individual food groups [75]. Understanding these relationships may be particularly important given the widespread consumption of dairy foods and the increasing interest in dietary recommendations for breast cancer survivors.

**FIGURE 5:**
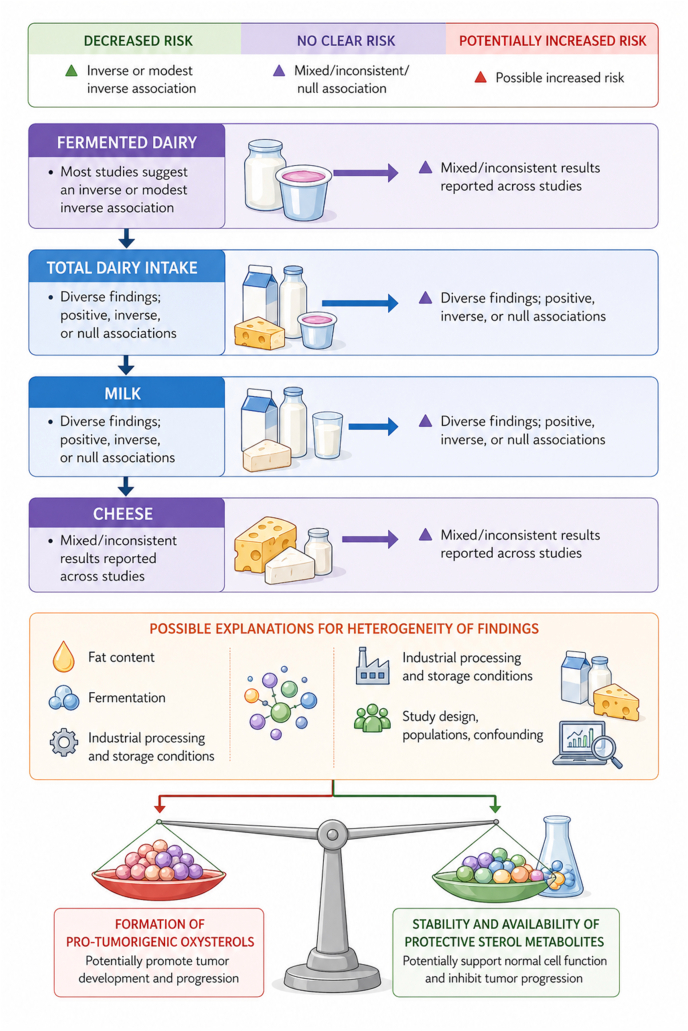
Summary of epidemiological associations between dairy consumption and cancer risk. This figure synthesizes findings from observational studies examining associations between dairy product consumption and cancer risk across different cancer types. Reported associations vary depending on the type of dairy product, level of processing, and population studied. These findings are subject to residual confounding, heterogeneity, and measurement limitations, and should not be interpreted as evidence of causality.

A summary of epidemiological associations between different dairy products and breast cancer risk is presented in Figure 5.

## Limitations of Current Literature

Interpretation of the epidemiological evidence on dairy intake and breast cancer is complicated by several methodological limitations affecting both exposure assessment and outcome classification. Although numerous cohort and case–control studies have examined this association, differences in study design, dietary assessment methods, and classification of dairy products make it difficult to draw firm conclusions.

One major limitation is the accuracy of dietary assessment. Most large cohort studies rely on food frequency questionnaires, which are subject to recall bias and measurement error. These tools often include only a limited number of items and typically classify dairy foods into broad categories such as milk, cheese, or yogurt, without distinguishing between different types of products or technological processing. Such imprecision may attenuate associations and contribute to inconsistent results across studies [76,77].

Another important limitation is the lack of detailed classification according to the degree of processing. Most epidemiological studies group dairy products into total dairy or major subcategories, without differentiating between minimally processed, fermented, processed, and ultraprocessed foods. However, industrial processing can substantially modify the nutritional and chemical composition of foods through heating, drying, addition of sugars or additives, and lipid oxidation. Because these changes may alter the biological activity of dairy products, failure to consider processing level may obscure true associations [69].

Confounding by overall dietary pattern and lifestyle factors also remains a concern. Individuals with different levels of dairy intake may differ in body weight, physical activity, alcohol consumption, and socioeconomic status, all of which are associated with breast cancer risk. Although most studies adjust for these variables, residual confounding cannot be excluded [[78], [79], [80]].

Another limitation is the limited number of studies examining tumor subtypes and prognosis. Because breast cancer is a heterogeneous disease, associations with dietary factors may differ according to hormone receptor status or molecular subtype, but only a few studies have analyzed these outcomes separately. Similarly, relatively few investigations have evaluated dairy intake after diagnosis, and most lack detailed information on the type and processing of dairy products.

In addition, most studies rely on self-reported dietary intake, which may not accurately reflect exposure to specific bioactive compounds generated during food processing.

A further limitation of the available epidemiological literature is the lack of standardized definitions of dairy intake categories. Terms such as "high," "higher," or "low" dairy intake frequently correspond to study-specific quantiles rather than harmonized consumption thresholds, limiting direct comparisons across studies.

Finally, most epidemiological studies focus on nutrient composition rather than chemical changes induced by food processing. Experimental studies indicate that heating and storage of lipid-rich foods can generate oxidized sterols and lipid peroxidation products with biological activity, but these compounds are rarely considered in dietary assessments [8,11,17,31,81]. Integrating information on food processing, chemical composition, and biological mechanisms may therefore be necessary to better understand the relationship between dairy products and breast cancer risk.

## Research Gaps and Future Directions

Despite the large number of epidemiological studies examining dairy intake and breast cancer, several important questions remain unresolved. Current evidence suggests that total dairy consumption is not consistently associated with breast cancer risk, although findings vary according to the type of dairy product, fat content, menopausal status, and tumor subtype. These inconsistencies highlight the limitations of conventional approaches that treat dairy foods as a homogeneous exposure.

One major gap concerns the role of food processing. Most epidemiological studies classify dairy intake into broad categories such as milk, cheese, or yogurt, without distinguishing between minimally processed, fermented, processed, and ultraprocessed products. However, technological processes such as heating, drying, fermentation, storage, and industrial formulation may alter food composition and generate oxidation products or other bioactive compounds. Although studies using the NOVA classification have reported associations between high consumption of ultraprocessed foods and increased cancer risk, processing categories alone may not adequately capture biologically relevant exposures. Products classified within the same category can differ substantially in their concentrations of lipid oxidation products, sterol-derived metabolites, additives, sugars, fatty acid composition, and other processing-related compounds. Consequently, the specific contribution of ultraprocessed dairy products remains insufficiently characterized [69,70].

Another limitation relates to the lack of data on chemical modifications induced by processing. Experimental studies indicate that heating and storage of lipid-rich foods can generate COPs and lipid peroxidation derivatives capable of modulating inflammatory and hormonal pathways [8,11,81]. These compounds may serve as precursors of sterol-derived metabolites involved in nuclear receptor signaling. However, direct evidence linking dietary exposure to such compounds with breast cancer risk in humans is currently lacking.

The role of endogenous sterol-derived metabolites also remains poorly understood. Some cholesterol-derived molecules, such as DDA, have been reported to exhibit tumor-suppressive properties in experimental models. However, their presence in foods, their stability during processing, and their relevance to human exposure remain uncertain. Similarly, although certain sterol oxidation products may contribute to the formation of metabolites with tumor-promoting properties in experimental systems, their occurrence in dairy products and their biological significance in humans have not been established.

The recent development of food composition databases including oxidized sterols, such as the FooDOxS database, provides new opportunities to address these questions [17]. However, coverage remains incomplete, particularly for processed dairy products, and data on specific sterol derivatives are still limited. Integrating detailed food composition data, lipidomic profiling, and prospective epidemiological studies may help clarify whether exposure to oxidized lipids or sterol-derived metabolites is associated with breast cancer risk or progression.

Another important consideration is the influence of metabolic context. Dietary patterns characterized by different macronutrient distributions, such as ketogenic diets, may alter systemic metabolism, including insulin and IGF-1 signaling, and could therefore modify the biological effects of dietary lipids [82]. However, evidence regarding the relationship between such dietary patterns and breast cancer in humans remains limited, and it is unclear whether the impact of dairy products differs according to metabolic state.

More broadly, current nutritional epidemiology relies largely on macronutrient-based assessments, which may not capture the biological activity of low-abundance molecules acting through signaling pathways. Future studies should therefore move beyond broad classifications such as “processed,” “ultraprocessed,” “fermented,” or “high-fat” dairy products and establish the actual composition of consumed dairy foods. In addition to assessing dairy intake, investigations should quantify serving-level exposure to COPs, POPs, epoxycholestanoids, lipid peroxidation products, fermentation-derived metabolites, additives, added sugars, and detailed lipid profiles. Such approaches may help determine whether observed associations are driven by the degree of processing itself or by specific molecular constituents generated during processing, storage, fermentation, or endogenous metabolism [17,31,81]. Overall, multidisciplinary approaches combining nutritional epidemiology, food science, and molecular biology will be required to evaluate these hypotheses and to determine whether processing-related changes in dairy composition are relevant to breast cancer risk and progression.

Although traditional approaches rely primarily on macronutrient composition, growing evidence suggests that low-abundance molecules, including lipid oxidation products and sterol-derived metabolites, may exert biological effects through signaling pathways. As highlighted in recent research on ultraprocessed foods, associations with health outcomes may persist even after adjustment for nutritional quality, suggesting that other components such as additives, processing-induced compounds, or contaminants may contribute to disease risk. Identifying these active substances, quantifying dietary exposure, and integrating this information into epidemiological studies will be essential to better understand the relationship between diet and cancer.

A key challenge for future research will be to move beyond nutrient-based approaches toward the identification of specific bioactive molecules present in foods or generated during processing. This shift may help explain why associations between diet and disease persist even after adjustment for nutritional quality, as suggested in recent studies on ultraprocessed foods.

In conclusion, the available evidence suggests that the relationship between dairy consumption and breast cancer cannot be fully explained by differences in nutrient composition alone. Variations in technological processing may influence the formation of oxidized lipids and other sterol-derived metabolites, which have been shown in experimental systems to interact with pathways involved in carcinogenesis.

However, the translation of these mechanisms to human populations remains uncertain. Current epidemiological studies do not account for exposure to oxidized lipids or processing-induced compounds, and the contribution of these factors to breast cancer risk has not been directly evaluated.

In line with emerging research on ultraprocessed foods, these observations support the hypothesis that compounds generated during food processing, beyond classical nutrients, may contribute to health outcomes. A major challenge for future research will be to identify, quantify, and characterize these bioactive molecules within complex food matrices, and to determine their relevance in real-life dietary exposures.

Integrating food composition databases, experimental models, and prospective cohort studies will be essential to bridge the gap between mechanistic insights and population-level evidence. Such approaches may help disentangle the respective roles of diet, metabolism, and food processing, and ultimately clarify whether specific compounds generated during dairy processing contribute to cancer-related pathways.

Although current evidence remains insufficient to establish causality, the identification of bioactive molecules formed during food processing, whether potentially deleterious or protective, represents a promising avenue for advancing our understanding of diet–cancer relationships and may help reconcile the heterogeneous associations observed in nutritional epidemiology.

This perspective aligns with emerging paradigms in nutritional epidemiology emphasizing the need to identify specific compounds driving associations beyond overall diet quality [15].

## Author contributions

The authors’ responsibilities were as follows – SA, JB, CG, LP, BA: contributed to writing – review & editing; PdM, MP, SS-P: contributed to conceptualization and writing – original draft; and all authors: read and approved the final version of the manuscript.

## Data availability

Data described in the manuscript, codebook, and analytic code will be made available on request pending application and approval.

## Declaration of Generative AI and AI-assisted technologies in the writing process

During the preparation of this work, the authors used ChatGPT (OpenAI) to improve the clarity and readability of the manuscript. After using this tool, the authors reviewed and edited the content as needed and take full responsibility for the content of the published article.

## Funding

This work was funded by an internal grant from the “Institut National de la Santé et de la Recherche Médicale,” the “Centre National de la Recherche Scientifique,” the “Université de Toulouse," the Institut National du cancer (PLBIO2020-028 and PLBIO2023-050), the Agence Nationale de la Recherche (DASYNT2, ANR-20-CE11-0005, DASYNT3, ANR-24-CE44-4332), the World Cancer Research Fundation (IIG_FULL_2024_015). We thank the charity associations “ELLES”, “J’y V,” Orange Care and URAFIKI JUU for their generous support.

## Conflict of interest

The authors report no conflicts of interest.
